# Identifying social outcomes of importance for childhood cancer survivors: an e-Delphi study

**DOI:** 10.1186/s41687-023-00676-7

**Published:** 2024-02-05

**Authors:** Sarah H. Milner, R. G. Feltbower, K. L. Absolom, A. W. Glaser

**Affiliations:** 1 Leeds Institute for Data Analytics, University of Leeds, Worsley Building, Clarendon Way, Woodhouse, Leeds, LS2 9NL UK; 2 Leeds Institute of Medical Research, University of Leeds, Leeds, UK; 3 Leeds Teaching Hospitals NHS Trust, Leeds, LS1 3EX UK

**Keywords:** Delphi technique, Survivors of childhood cancer, Consensus, Outcomes

## Abstract

**Purpose:**

Childhood cancer survivors (CCS) are at risk of deficits in their social outcomes, a key aspect of overall health and quality of life. Social outcomes of import are ill-defined leading to potential gaps in research and service provision. In this study, we undertook a preliminary consensus seeking exercise to support the development of a framework of the important social outcomes for CCS.

**Methods:**

A modified e-Delphi study was conducted with four groups: CCS, health professionals, social workers and teachers. Round 1, developed from a literature review, included 34 questions rated for importance on a 7-point Likert scale. Rounds 2 and 3 presented items not achieving consensus, additionally proposed items and in round 3, a ranking question.

**Results:**

Survey 1 was completed by 38 participants, 31 (82%) completed survey 2 and 28 (76%) completed survey 3. A total of 36 items were prioritised across 6 domains (education, independence, work, relationships, community, lifestyle), together forming the final list of social outcomes. Of these, 22 items met consensus for importance. Items rated most important were “having autonomy” and “avoiding social isolation”. Quantitative and qualitative results reflected that social outcomes for survivors and general public should be the same.

**Conclusion:**

We have generated initial consensus on important social outcomes for CCS, highlighting the need for these to be matched to those of the general population. It suggests strategies are required to ensure autonomy and appropriate support for independence and relationships are provided through long-term aftercare and beyond. Further work is needed to validate and develop these findings into a framework to support appropriate social aftercare for CCS.

**Supplementary Information:**

The online version contains supplementary material available at 10.1186/s41687-023-00676-7.

## Abstract (plain language summary)

Following treatment for childhood cancer, survivors may face problems with their social health. These are the parts of life, besides physical and mental health, that help people to lead full, happy and satisfied lives. Social health is important as it affects all areas of our lives and includes many areas such as education, work and relationships. It is essential that we understand what the most important areas of social health are for childhood cancer survivors so that we can support these. This will help survivors lead the lives they want as adults. In this study, 38 childhood cancer survivors, children’s cancer doctors and nurses, social workers and teachers took part in a series of questionnaires designed to collect their views about social health for survivors. Participants established 22 areas of social health to be very important. ‘Having autonomy’ and ‘avoiding social isolation’ were selected as the most important. Participants felt that the aspects of social health which are important to survivors are the same outcomes as for the wider public. However, survivors may face many barriers to doing well in these areas. To fully identify the important areas of social health for childhood cancer survivors, we need to undertake further work to understand the views of other important groups, such as parents or carers. We also need to explore the barriers survivors face in achieving good social health.

**Supplementary Information:**

The online version contains supplementary material available at 10.1186/s41687-023-00676-7.

## Introduction

Recent decades have seen numerous advancements in detection, treatment and supportive care of children with cancer leading to increased survival rates. In the United Kingdom (UK), this is now 86% [1, 2]. Alongside an increasing incidence of childhood cancer globally [3], this means that more children than ever before are becoming adult survivors with over 35,000 in the UK [4] and 500,000 across Europe [5].

Childhood cancer survivors (CCS) face numerous long-term effects of their disease and treatment, with this burden increasing with attained age [6]. Effects are seen across all areas of the biopsychosocial model of health [7] with much known about the physical impacts [6] but more work is needed to fully explore psychosocial outcomes [8].

‘Social outcomes’ is a broad term and because a number of its likely constituents are subjective in nature, it is difficult to accurately categorise. It could be described as ‘a diverse set of social capacities linked to personal functioning and functioning in social structures such as the labour market, social groups and society’ [9].

CCS may experience impacts across various social outcomes including: level of educational attainment [10–12], occupational outcomes including employment status and salary [12–14], independence and autonomy [15, 16]and relationships including friendships and intimate relationships [17, 18].Survivors have lower rates of marriage or cohabitation than the general population [19, 20] and social isolation may be a problem [21]. Impacts are greatest following CNS disease or cranial radiotherapy [10–20].

The World Health Organisation (WHO) defines health as a ‘state of complete physical, mental and social well-being and not merely the absence of disease or infirmity’ [22]. This demonstrates the complex interactions between components of health and the need to understand and promote positive social outcomes required to achieve the best health possible in CCS. Social health is also critical in achieving good Quality of Life (QoL). Positive social outcomes are fundamental to several domains within the WHO’s QoL assessment tool: level of independence, social relationships, environment and spirituality/religion/ personal beliefs of the WHO’s QoL assessment tool [23]. In addition, deficits in two other domains: physical health and psychological may represent some of the barriers to achieving a good social outcome for childhood cancer survivors further demonstrating the closeness of their relationship [23].

Despite the widely-accepted WHO definition of health, the use of self-reported QoL measures and research revealing potential deficits in social outcomes, there is not an appreciable body of work to generate useful, meaningful indicators of social outcomes for CCS. This is needed to guide future research and, ultimately, finite service provision to reduce social inequity experienced by survivors.

Delphi methodology’s ability to connect the views of different stakeholders and experts to achieve consensus opinion across multiple health research contexts [24, 25] makes it an appropriate tool to address issues benefiting from collective, subjective judgement such as understanding the important social outcomes for CCS. A preliminary study utilising this method was determined to be an important first step in generating consensus regarding social outcomes of import for CCS and subsequently a framework to provide appropriate support to achieve optimal outcomes.

### Objective

To explore the social outcomes of importance for CCS using a modified e-Delphi technique.

## Methods

The protocol is fully described elsewhere [26]. However, in brief, a modified e-Delphi methodology was followed, incorporating recognised components including: (i) use of an ‘expert’ panel (ii) anonymity of panel members, (iii) iterative rounds of survey and controlled feedback and (iv) iteration until consensus is achieved [25]. Three rounds of surveys were conducted via Online Surveys (www.onlinesurveys.ac.uk) from 08/03/2022 to 25/07/2022. The initial survey was developed from a literature review with input from a CCS, teacher and nurse. The questions were further refined following cognitive testing with potential participants. Participants were asked about a variety of different areas in 6 key categories (education, independence, work, relationships, community life and lifestyle) to identify what outcomes might be important.

### Participants

The study recruited four ‘expert’ groups: CCS, paediatric oncology doctors and nurses, teachers, and social workers experienced in supporting young people living with and beyond childhood cancer. To be eligible, CCS needed to be: 18 years of age or older, have a previous cancer diagnosis before age 18 and be 5 years or more from treatment completion.

CCS were identified purposively from the long-term follow-up (LTFU) service at Leeds Teaching Hospitals NHS Trust (LTHT), a regional children’s cancer principal treatment centre (PTC) in the north of England and approached in writing. The purposive strategy ensured a range of diagnoses, genders and ages were included. Health professionals were recruited via the Children’s Cancer and Leukaemia Group (CCLG), an organisation for paediatric oncology professionals in the United Kingdom (UK) [27]. Invitations were sent to members via e-newsletter and verbally at a CCLG Late Effects Special Interest Group meeting. Teachers and social workers were recruited at 2 regional children’s cancer PTCs. Both groups had experience supporting children with cancer e.g. teachers at the hospital school associated with the PTC were approached. Managers of the two groups identified potential participants and made initial contact for the study team. Individuals from other regions could participate provided they met eligibility criteria.

Interested individuals contacted the study team by email or telephone. Email addresses for individuals willing to participate were stored on a secure University of Leeds server. Links to surveys were distributed by email. Up to four reminders were sent per round.

### Sample size

There is no definitive, optimum number of participants for a Delphi study [28]. Previous recommendations include a minimum total of 12 [29]. 80 patients were approached in writing. The exact number of professionals approached is unknown given the sampling strategy.

### Analysis

Medians were used to summarise response scores and determine the importance of items. Items achieving a median of 6 or 7 on a 7-point Likert scale were defined as “important” or “very important” and were immediately included in the definitive list of outcomes. Items scoring < 6 were re-presented in the subsequent round for review. If an item’s median did not change after re-presentation, it was not presented again. Interquartile range (IQR) and mean average deviation from the median (MADM) were calculated as indicators of strength of agreement. All scores were calculated according to group (patients, professionals). Free text comments underwent content analysis [30] to identify whether they supported the median scores for each item and to identify any new themes relevant to the work.

### Procedure

*Round 1 survey* distributed to 50 registered individuals in March 2022, remaining open for 3 weeks. Consent, age group (18–34, 35–44, 45 +), ethnicity and gender were collected. Participants were asked to: (1) rate the importance of 6 categories and 34 subcategories of social outcomes on a Likert scale from 1 (not at all important) to 7 (very important) for CCS and the general public separately; (2) provide reasons for their ratings and (3) suggest additional categories. CCS and the general public were asked to separately to (a) encourage non-survivor participants to consider CCS as a distinct group and (b) investigate whether outcomes for both groups ought to be the same. See Additional file 1 for all questionnaires.

*Round 2 survey* beginning in May 2022, this ran for 4 weeks and was distributed to the 37 individuals completing round 1. 12 categories from round 1 were re-presented alongside 5 new categories. Each participant was shown their previous score and the group median on personalised Likert scales with results of the qualitative analysis to aid their decision making.

*Round 3 survey* beginning in June 2022 and running for 4 weeks, this was distributed to the 31 individuals completing round 2. Three categories from round 2 were presented for re-rating in the same way as round 2. Participants were asked to select their three most important items from the list of 22 subcategories achieving scores of 6 or 7.

### Ethics

Approval for the study was granted by the NHS Health Research Authority’s Regional Ethics Committee 4, West of Scotland (ID 297344).

## Results

A total of 50 individuals registered as panel members, of whom 37 (74%) completed the first-round questionnaire. Respondent characteristics for each round are reported in Table 1 (characteristics not broken down into professional and patient groups due to small numbers and consequent risk of identification). In rounds 2 and 3 respectively, 31/37 (84%) and 28/31 (90%) responded, providing a response rate of 75% overall. In the CCS group, a range of disease types were represented including brain and spinal tumours (3), leukaemias (3) and other solid tumours including lymphomas, sarcomas, Wilm’s tumour and neuroblastoma (8). Treatment of the patients participating included chemotherapy, radiotherapy, surgery and stem cell transplant.

**Table 1:** Characteristics of panel members who participated in Rounds 1–3

All participants	N (total)	Round 1	Round 2	Round 3
		n = 37	n = 31	n = 28
Childhood cancer survivors	N	14	12	12
Professionals	N (total)	23	19	16
	Health professionals	12	11	10
	Teachers	5	3	2
	Social workers	6	5	4
Age (all participants)	18–34	10	9	8
	35–44	8	6	6
	45+	19	16	14
Gender (all participants)	Male	9	9	9
	Female	28	22	19
Ethnicity (all participants)	White British	33	27	24
	Any other ethnic background	4	4	4

### Round 1 (Table 2)

**Table 2:** Quantitative results from Round 1 (n = 37)

Item		Patient participants (n = 14)						Professional participants (n = 23)						All participants (n = 37)												Outcomes
														General public						Childhood cancer survivors						
		Median		IQR		MADM		Median		IQR		MADM		Median		IQR		MADM		Median		IQR		MADM		
*Education*																										
A. Receiving an education		7		0.75		1.00		7		0		0.35		7		1		0.41		7		0		0.59		Accepted
B. Complete school up to age 18		6		1.75		0.86		7		1		0.70		7		1		0.65		7		1		0.81		Accepted
C. Complete vocational training		5.5		1.75		1.07		7		1		0.48		7		1		0.76		6		1		0.84		Accepted
D. Complete higher education		4.5		2		1.36		5		2.5		1.10		5		2		0.97		5		3		1.19		Re-presented
E. Good school attendance		6.5		2.75		1.71		6		2		1.13		7		1		0.51		6		2		1.35		Accepted
F. Good exam grades		5		1.5		1.07		5		1		0.74		5		1		0.76		5		1		0.86		Re-presented
G. Have enjoyed school		6		3		1.79		6		1		0.87		5		2		1.24		6		2		1.22		Accepted
H. Participated in extra-curricular activities		6.5		3		1.78		6		1.5		1.00		5		2		1.08		6		3		1.30		Accepted
*Independence and autonomy*																										
I. Live independently		6.5		1		0.71		7		1		0.57		7		1		0.68		7		1		0.62		Accepted
J. Have autonomy		7		0		0.29		7		0		0.17		7		< 1		0.11		7		0		0.22		Accepted
K. Undertake big responsibilities e.g. children		6.5		1.75		0.93		6		2.5		1.22		6		2		1.08		6		2		1.11		Accepted
*Work and finances*																										
L. Financial stability		7		1		0.50		7		1		0.39		7		1		0.46		7		1		0.43		Accepted
M. Be able to save for luxuries		5.5		1.75		1.00		5		1		0.74		5		1		0.76		5		2		0.84		Re-presented
N. To have a job		7		0.75		0.57		6		1		0.61		7		1		0.65		7		1		0.68		Accepted
O. Well paid job		7		1		1.00		6		1		0.57		6		1		0.76		6		1		0.84		Accepted
P. Job satisfaction		6		1.75		0.93		6		1		0.48		6		1		0.65		6		1		0.65		Accepted
Q. Job that builds skills		5		1		0.79		6		2		0.83		6		2		0.92		6		2		0.92		Accepted
R. Be a homeowner		4		2.75		1.43		4		1		0.83		4		1		1.08		4		1		1.05		Re-presented
S. Doing better than others financially		3.5		2		1.29		3		2		1.22		3		2		1.22		3		2		1.24		Re-presented
*Relationships*																										
T. Having relationships with others		7		0		0.29		7		0.5		0.39		7		1		0.38		7		0		0.35		Accepted
U. Good family relationships		7		0		0.34		7		1		0.74		6		1		0.76		7		1		0.59		Accepted
V. Good friendships		7		1		0.86		7		1		0.61		6		1		0.78		7		1		0.70		Accepted
W. Good romantic relationships		5		2		1.29		5		1		0.65		5		1		0.89		5		1		0.89		Re-presented
X. Good work relationships		5.5		2		1.14		6		1.5		0.74		6		2		0.89		6		2		0.89		Accepted
Y. Communicate with formal organisations e.g. for benefits		4		1		1.07		5		2		1.04		5		2		1.19		5		2		1.16		Re-presented
Z. To avoid social isolation		7		1		0.50		7		1		0.43		7		1		0.65		7		1		0.46		Accepted
*Community life*																										
AA. Local community involvement		4		2		1.21		5		2		1.01		4		1		1.22		5		2		1.22		Re-presented
BB. Take part in community activities		4		2.75		1.36		5		1.5		0.96		5		2		1.14		5		2		1.16		Re-presented
CC. Support others e.g. charity work		5		2.5		1.21		4		1		0.96		4		1		0.97		4		1		1.11		Re-presented
DD. Being able to participate in a religious or spiritual community if desired		4		4.75		2.07		5		3		1.21		5		3		1.68		5		3		1.65		Re-presented
EE. Social identity		4.5		3.75		1.93		5		1		1.00		5		2		1.32		5		2		1.35		Re-presented
*Lifestyle*																										
FF. Personal maintenance		7		0		0.21		7		0.5		0.30		7		1		0.35		7		0		0.27		Accepted
GG. Positive lifestyle choices e.g. exercise	7		0.75		0.43		7		1		0.61		7		1		0.73		7		1		0.54		Accepted	
HH. Avoid risky health behaviours e.g. smoking	7		0.75		0.50		7		1		0.78		6		2		0.92		7		1		0.68		Accepted	

The median, interquartile range and mean absolute deviation from the median are presented for each item listed in the round 1 survey for all participants, the patient group and the professionals’ group in the context of childhood cancers survivors. For the “all participant group” the scores given for the general public for comparison are presented. For full descriptions of all items, see Additional file 1

A total of 22 items had medians of 6 or 7, meeting criteria to be included as important social outcomes. A further 12 items had medians between 3 and 5 necessitating re-presentation in round 2. No items were removed. An additional 5 items were suggested by participants: *‘being able to have sexual relationships’, ‘being able to access peer support…’, ‘being able to drive’, ‘being able to access and utilise public transport’ and ‘being able to express yourself creatively…’*. See Additional file 1 for full statements. Of the 22 items meeting ‘important’ criteria, 18 had IQRs and MADM of < 1. The highest IQRs and MADMs were seen in the category ‘community life’. CCS had higher overall IQRs and MADMs than the professional group.

All medians for CCS and the general public were the same or within one Likert scale point of each other. Free text comments strongly supported this with a clear theme that important outcomes for survivors and the public should be the same. See Additional file 2 for summary comments.

Score variability across all participants and a large majority (31/37) making at least one comment alongside their numerical score demonstrated good engagement. See Table 2 for Round 1 results.

### Round 2 (Table 3)

**Table 3:** Quantitative results from Round 2 (n = 31)

Item	Patient participants only (n = 12)			Professional participants only (n = 19)			All participants (n = 31)				Outcome
	Median	IQR	MADM	Median	IQR	MADM	Median	IQR	MADM	Change in score	
*Education*											
D. Complete higher education	4.5	1.75	1.25	5	1	0.63	5	2	0.87	No	Accepted- no score change
F. Good exam grades	5	0.75	1.00	5	1	0.58	5	1	0.74	No	Accepted- no score change
*Work and finances*											
M. Save money for luxuries	5	1.25	0.75	5	1	0.58	5	0	0.55	No	Accepted- no score change
R. Be a homeowner	4	1.25	0.83	4	1.5	0.74	4	1	0.68	No	Accepted- no score change
S. Doing better than others financially	3	1.25	0.83	3	0.5	0.42	3	2	0.77	No	Accepted- no score change
*Relationships*											
W. Good romantic relationships	5	1.00	1.00	5	1	0.58	5	1	0.74	No	Accepted- no score change
Y. Communicate with formal organisations e.g. for benefits	5	1.25	0.75	5	1	0.68	5	1	0.71	No	Accepted- no score change
*Community life*											
AA. Local community involvement	4.5	1	0.67	5	1.5	0.63	5	1.5	0.65	No	Accepted- no score change
BB. Take part in community activities	5	1	0.67	5	0.5	0.47	5	1	0.55	No	Accepted- no score change
CC. Support others e.g. charity work	4.5	1	0.67	4	1	0.68	4	1	0.68	No	Accepted- no score change
DD. Being able to participate in a religious or spiritual community if desired	4	1.5	1.67	5	2	0.95	5	2	1.22	No	Accepted- no score change
EE. Social identity	5	2.25	1.33	6	1	0.68	5	1	0.94	No	Accepted- no score change
*Newly suggested items*											
FF. Sexual relationships	5	1.25	1.00	5	2	0.79	5	2	0.87	N/A	Re-presented
GG. Access peer support	5	2.25	1.25	6	2	0.95	6	2.5	1.06	N/A	Accepted
HH. Being able to drive	4.5	3.5	1.75	5	1	0.74	5	1.5	1.13	N/A	Re-presented
II. Access public transport	6	1.25	0.74	6	1.5	0.84	6	1.5	0.81	N/A	Accepted
JJ. Being able to express yourself creatively	5	2	1.00	5	2	1.00	5	2	1.00	N/A	Re-presented

The median, interquartile range and mean absolute deviation from the median are presented for each item. Items are made up of those with medians ≤ 5 in round 1 and items newly suggested by participants. For full descriptions of all items, see Additional file 1

No re-presented items achieved medians of 6 or 7. All maintained their original scores and were not presented again. Of the newly presented items, 2, *‘being able to access peer support…’ and ‘being able to access and utilise public transport’* had medians of 6 meeting criteria to be included as important. Three remaining items: *‘being able to have sexual relationships’, ‘being able to drive’ and ‘being able to express yourself creatively…’* required re-presentation. No items were removed, and no further items were suggested. The IQR and MADM were lower compared to round 1 in re-presented categories, particularly in the category ‘community life’. All participants had score variability and all made changes to their scores and/or described their rationale. See Table 3 for Round 2 results.

### Round 3 (Table 4)

**Table 4:** Quantitative results from Round 3 (n = 28)

Item	Patient participants only (n = 12)			Professional participants only (n = 16)			All participants (n = 28)				Outcome
	Median	IQR	MADM	Median	IQR	MADM	Median	IQR	MADM	Change in score	
AF. Sexual relationships	5	1	0.75	5	1	0.56	5	1.25	0.64	No	Accepted—no score change
AH. Being able to drive	5	1.25	1.00	5	0	0.38	5	1	0.64	No	Accepted—no score change
AJ. Being able to express yourself creatively	5	2	0.92	5.5	2	0.88	5	2	0.89	No	Accepted—no score change

The median, interquartile range and mean absolute deviation from the median are presented for each item. Items are made up of those which were newly suggested in round 1 and had a median ≤ 5 in round 2. For full descriptions of all items, see Additional file 1

No re-presented items achieved medians of 6 or 7. All maintained their original scores. The IQR and MADM were lower for *‘being able to have sexual relationships’ and ‘being able to drive’*. All participants made changes to their scores and/or described their rationale. See Table 4 for Round 3 results.

**Fig. 1:**
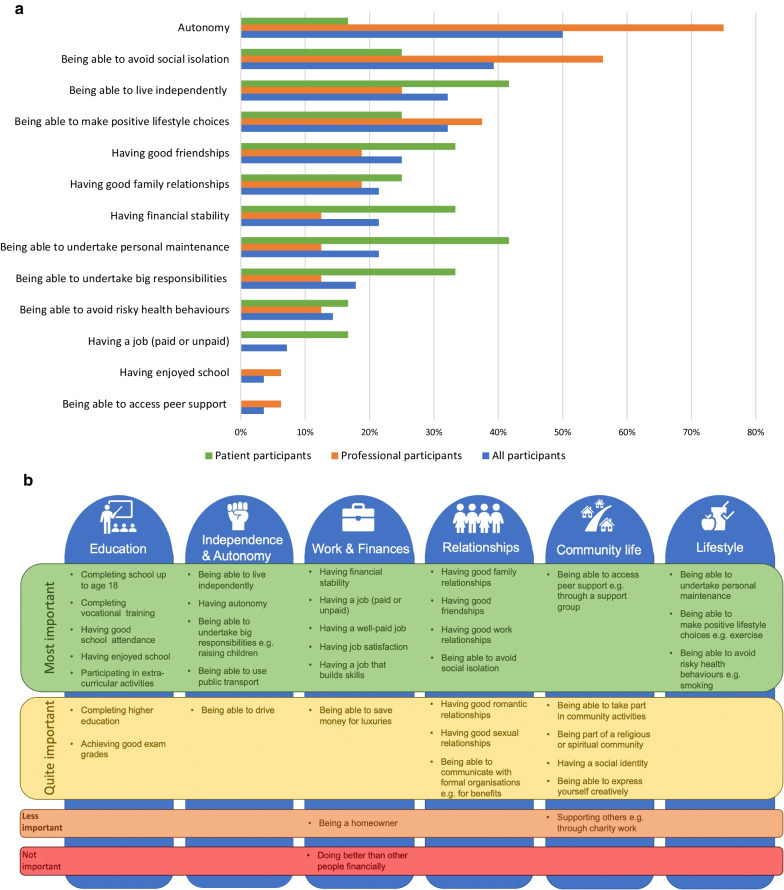
**a** Percentage of participants selecting high scoring items (median of 6 or 7) in their ‘top 3’ overall. Only those items ranked by any participants are displayed. **b** Final list of social outcomes from most to least important across the 6 major categories: education, independence and autonomy, work and finances, relationships, community life and lifestyle

Participants were asked to rate their top three items from the list of 22 achieving a median of 6 or 7. The five items rated as top three by the most participants were ‘*having autonomy’* (14 participants, 50%), ‘*being able to avoid social isolation’* (11 participants, 39%), ‘*being able to live independently*’ (9 participants, 32%), ‘*being able to make positive lifestyle choices’* (9 participants, 32%) and ‘*having good friendships*’ (7 participants, 25%). Over 50% of participants rated an aspect in the ‘lifestyle’ category in their top 3. When broken down by group, professionals rated ‘*having autonomy’* highest, whereas patients selected ‘*being able to live independently’* and ‘*being able to undertake personal maintenance*’. Nine items were not chosen by any participant: ‘*having a job that builds skills’, ‘having good school attendance’, ‘having good work relationships’, ‘having a well-paid job’, ‘being able to undertake extra-curricular activities’, ‘completing school up to 18’, ‘having job satisfaction’, ‘being able to complete vocational training’ and ‘being able to use public transport’*. Figure 1a displays these results.

At the end of the study, the final list of items was shown to participants for comments. All participants indicated their agreement. It comprised 6 major categories: education, independence and autonomy, work and finances, relationships, community life and lifestyle. Across all categories, 22 items were deemed most important, 11 as quite important, 2 as neither important nor unimportant and 1 as not important. These were derived from the median scores. Figure 1b displays the final list.

Free-text comments provided the rationale for participant scores throughout the process. They were supportive of themes which emerged from the median scores and ranking question and provided depth. See Additional file 2 for the summary.

## Discussion

To our understanding, this study is the first systematic attempt to work towards developing a consensus on the important social outcomes for CCS. It has demonstrated the use of the Delphi method for generating a definitive social outcomes framework. The top 5 outcomes were: ‘*having autonomy’*, ‘*being able to avoid social isolation’*, ‘*being able to live independently*’, ‘*being able to make positive lifestyle choices’* and ‘*having good friendships*’.

Assessment of social outcomes can, in part, be based on self-reported QOL/ Patient Reported Outcome Measures. In addition, linkages between cancer registry data and administrative datasets (e.g. education, tax and benefits) can also provide valuable insight on how CCS are functioning in everyday life. However, a clear definition of the indicators which most meaningfully provide insight on social outcomes following childhood cancer are lacking. This work initiates the path towards defining this.

A key finding was that the important social outcomes for CCS and the general public ought to be viewed as being the same. We are not aware of this concept being studied before, however it does align with the desire to ‘feel normal again’ and move forward with life which is well documented [31]. This understanding contributes to the knowledge that we must strive for a normal social trajectory for survivors. It may help families and professionals to promote the return of normal boundaries as soon as practically and emotionally possible for the family. Survivors may face barriers to achieving this desired trajectory [32, 33] which need to be understood and addressed if outcomes are to be matched to those of the general public. Addressing these barriers would appear to be an important role for multi-disciplinary teams during cancer care and subsequently during the aftercare pathway.

Autonomy and independence are key aspects of adult life with independence being a primary domain in the WHO QoL instrument [23]. Autonomy may pertain to health and other aspects of life. The myriad of late effects that evolve across the CCS’ lifetime make it critical that they understand their health needs and are able to make relevant, informed decisions for themselves. Engaging CCS more in their healthcare should help facilitate this, promoting greater adherence to healthcare recommendations and better health-related quality of life [34]. Survivors have reported feelings of increased personal maturity including autonomy compared to peers which they see as both positive and negative. CNS tumour survivors conversely experience lower rates of autonomy, which is potentially explained by some of the cognitive impacts they may face [35].

Professionals rated autonomy most highly whilst CCS marking independence as the top priority. Although the concept of autonomy was explained as part of the item description, and the survey piloted for understanding, it might be more familiar to professionals than patients. It is unclear whether CCS truly value independence over autonomy: more research is required to address this. Financial independence is a crucial aspect of overall independence. Survivors experience occupational deficits, including health-related unemployment [14], risking this independence. Ongoing work is necessary to support survivors to access and maintain appropriate employment.

The importance of social relationships and the risk of social isolation are well recognised [36, 37] and, as for independence, a primary domain in the WHO QoL instrument [23]. Participants rating *‘avoiding social isolation’* and *‘having good friendships’* highly reflects this. CCS risk interruption to normal relationship trajectories and may experience social isolation [12, 21, 38]. Contributing factors include age at diagnosis and treatment, sex, diagnosis, treatment intensity, socioeconomic status, cognitive and physical impairments and poor body image [39]. Interestingly, despite social isolation being rated highly, community activities, which would reduce the risk of social isolation, were felt to be less important. Knowledge of the importance of these outcomes should encourage clinicians to screen for isolation, particularly in those with risk factors such as cognitive and physical impairment. A rapid screen could be performed by repurposing a research tool such as the Steptoe Social Isolation Index [40] or the Three-Item UCLA Loneliness Scale [41]. Development of therapeutic programmes, such as peer support/ training groups (which rated highly in this study), has the potential to facilitate peer-to-peer connection, decrease isolation and promote a sense of normality [42].

The ‘top 3’ priority setting question within the final round was deemed necessary given the many items designated important by participants. Finite resources make identifying priority areas important. Although there was some overlap in the most important areas selected by patients and professionals, they did not completely align. Fundamentally, the voice of the patient must be paramount, but it is of interest to understand the views of professional groups. The disagreement may be in part due to the different experiences of the cancer journey between the groups: Professionals have experience of different stages of the survivorship journey across multiple patients whereas patients can only report on their own lived experience to date which, whilst being in great depth, inevitably relates to them alone and is limited to the life-stage they have attained at the time of the assessment. Qualitative work to explore these disagreements in more depth may yield important messages.

Comprehensive and holistic LTFU care focussing on more than physical health outcomes is necessary if all survivors are to be supported to achieve social success in the areas designated as important in this study. National and international LTFU care recommendations include guidance on psychosocial evaluation in all survivors, particularly those at highest risk [43, 44]. However, it is unknown how well this is covered during clinical episodes nor whether all clinicians have access to appropriate support for their patients should needs be identified. Furthermore, CCS may be designated as ‘low risk’ for late effects with care transferred to the community. This ‘low risk’ stratification is invariably based on the risk of physical health adverse sequelae with little regard to the risk of psychosocial late effects. Whilst being deemed to be ‘low risk’, they may still experience deficits in social outcomes making it equally important that General Practitioners and community health care professionals have knowledge and access to social support options.

## Strengths and limitations

The main strengths of our work are that it is based on the responses of a range of CCS with varied diagnoses and professionals with experience of childhood cancer in different settings and different stages of the cancer journey. The varied diagnoses ensured that participants with a broad spectrum of long-term complications including physical disabilities were included. There was good retention (75%) and good engagement. We have published the study protocol in line with recommendations encouraging transparency in the Delphi process [45].

There are several limitations. The sampling strategy, whilst chosen to ensure appropriate representation of those individuals thought to be at greatest risk of experiencing the most extreme deficits in social outcomes, will have introduced selection bias. It facilitated inclusion of CNS tumour survivors, a group often excluded from qualitative research, yet we recognise that those with greatest cognitive impairment were still unable to participate. Carers, who could provide insights into this group as well as their own, unique perspective of the cancer journey were not involved. Participants were restricted to UK residents and English speakers, reducing potential generalisability. Limited ethnic diversity (92% white) may mean that key differences in viewpoint have been missed from minority ethnic groups who already experience disparities in health outcomes [46]. There was an unequal gender divide although this reflects the make-up of the workforce in some of these areas. The desired minimum of twelve participants per group was not achieved but all planned groups had representation and the medians between the groups were within one Likert scale point. The sampling strategy for professional groups and indirect contact prevented any risk of feeling pressured to take part but meant there was no way of knowing who had received invitations, potentially affecting recruitment. Attempts were made to understand the low recruitment in some professional groups, with service pressures being the primary reason given.

### What next?

Further work with individuals from varied backgrounds will be key to building the most comprehensive picture of important social outcomes for survivors. This requires a large-scale study which must include CCS from ethnically diverse backgrounds, those with known cognitive and/or physical impairment including visible physical sequelae, those with mental health concerns and carers. Fully understanding and maximising social outcomes in a rapidly changing social environment is complex. Despite the limitations of this work, it is a step towards building a framework to help monitor, measure and ultimately improve social outcomes for this population [47]. The next phases of this work, including semi-structured interviews with CCS and carers, will help more closely examine the specificity of the framework’s relevance for this group. Finally, administrative datasets will have the ability to provide great insight into how CCS are functioning in the areas of social outcomes defined by this work. Exploring CCS’ views of using this data would therefore be another important step.

## Conclusion

CCS should strive for autonomous, independent lives, with social goals and ambition matched to the general population. The research has re-affirmed the importance of relationships and the need to support at-risk survivors to avoid social isolation. It has highlighted clear areas for further work in addition to the limitations which must be addressed when developing the methodology for the definitive consensus generating study. Finally, findings emphasise the need to further explore barriers to achieving success across key areas of social function to help maximise the overall health of each person living with and beyond childhood cancer.

### Supplementary Information

**Additional file 1: **Round 1-3 Questionnaires for all participants.

**Additional file 2: **Qualitative results overview.

## Data Availability

The datasets generated and analysed during the current study are not publicly available as individual privacy could be compromised by the demographic information provided alongside detailed free-text comments.
