# Supplementary material for: Identifying social outcomes of importance for childhood cancer survivors: an e-Delphi study
Source: J Patient Rep Outcomes. 2024 Feb 5;8:14. doi: 10.1186/s41687-023-00676-7 (PMC10844160; doi:10.1186/s41687-023-00676-7)
Supplement: Supplementary file 1 — Additional file 1: Round 1-3 Questionnaires for all participants. [file 41687_2023_676_MOESM1_ESM.docx]

# Identifying Social Outcomes of Importance for Childhood Cancer Survivors: An e-Delphi Study. Supplementary File 1.

**ROUND 1-3 QUESTIONNAIRES, ALL PARTICIPANTS**

**ROUND 1 QUESTIONNAIRE**

WELCOME

**Thank you for agreeing to take part in this study.**

**Firstly, we need to confirm that you have had information about the study and consent to be involved.**

Please answer these questions to confirm that you have had information about the study and consent to take part.

- 1. I have read the participant information leaflet yes/no
  2. I understand how my answers will be used yes/no
  3. I consent for my email address to be stored securely at the University of Leeds for the purpose of this study yes/no
  4. I consent to take part in this study yes/no

1. Please choose the participant group you fall into (Click box leads to further question based on first box chosen)
   1. Patient -> Leukaemia/Brain or spinal tumour/ Other cancer
       -> Age 18-29years / >29 years
   2. Clinical-> Doctor/ nurse
   3. Social worker
   4. Teacher-> Primary/secondary
       -> State/ Fee paying
2. We would like to collect some basic information that will help us to understand who is taking part in the study:
   1. Please choose one option that best describes your ethnic group or background: (As per census ethnic groups)

- White: English, Welsh, Scottish, Northern Irish or British; Irish; Gypsy or Irish Traveller; Any other White background
- Mixed or Multiple ethnic groups: White and Black Caribbean; White and Black African; White and Asian; Any other Mixed or Multiple ethnic background
- Asian or Asian British: Indian; Pakistani; Bangladeshi; Chinese; Any other Asian background
- Black, African, Caribbean or Black British: African; Caribbean; Any other Black, African or Caribbean background
- Other ethnic group: Arab; Any other ethnic group
- Prefer not to say
  1. Please choose one option that best describes your gender
- Female
- Male
- Prefer to self-describe: ……………………………………….
- Prefer not to say
  1. Please select your age bracket
- 18-24, 25-34, 35-44, 45-54, 55-64, 65+ & ‘Prefer not to say’

- What is leading a good life for childhood cancer survivors?

Leading a good life is more than having good mental and physical health alone. There are other aspects of life that are needed to ensure happiness, satisfaction and a sense of fulfilment – allowing a person to be the most that they can be. We think of these as 'social health'.

We are interested in what you think these other things are for adults and which of them are the most important.

We are particularly interested in seeing whether you think they are the same for adults who had cancer as a child and adults in the general population. 

All the responses we get will be brought together over the rounds of the project. We will use the results to target research in the important areas so that in the future we can provide the right support to survivors when they most need it. 

- SCORING

Over the following pages, we are going to ask some questions about how important different things are for adults who had cancer as a child and the general, adult population.
 
Please rate each idea from 1 to 7.

****1**** is ****NOT at all important**** and ****7**** is ****VERY important****.

In the text box below each idea, please write down any reasons for your rating. This will help us to understand your answers and prepare for the next round of questions. 

We also want to know of any other ideas you have and will ask this at the end.

For all of the following questions there is an individual 7 point Likert scale for adults who had cancer as a child and for the general adult population with a free-text box under each when viewed one the survey host website: [www.Onlinesurveys.ac.uk](http://www.Onlinesurveys.ac.uk).

See appendix 1 for example as seen on Online Surveys.

1. Education

   This section looks at whether education is or has been important. For childhood cancer survivors, this includes any point from diagnosis.

   A. How important is it to receive an education? This is being able to go to school, college and university for example.
2. How important is it to complete school up to age 18?
3. How important is being able to complete vocational training e.g. NVQs or apprenticeships?
4. How important is being able to complete higher education e.g. getting a degree at university or similar?
5. How important is it to have good school attendance as a child?
6. How important is it to achieve or have achieved good exam grades?
7. How important is it to have enjoyed school?
8. How important is it to have participated in extra-curricular activities such as sport or music clubs?
9. Independence and Autonomy

   Next, we’re going to think about independence and autonomy.
   1. How important is being able to live independently?
10. How important is it to have autonomy? This is a person being able to make their own decisions about how they want to live.
11. How important is being able to take on big responsibilities such as raising a child?
12. Work and Finances

    This section looks at work and finances
13. How important is it to have financial stability? This is a person being in control of their day-to-day finances and having enough money to meet their needs including saving for retirement or emergencies.
14. How important is it to be able to save money for luxuries? These might include holidays abroad, meals out or a better car than necessary.
15. How important is it to have a job? This could be paid or unpaid.
16. How important is it to have a job that pays well enough to be independent and do the things a person wants to do?
17. How important is it to have job satisfaction? This means a person feeling content with their job and getting fulfilment from it.
18. How important is it to have a job that builds skills?
19. How important is it to be a homeowner? This means owning a home rather than renting. It could be with or without a mortgage.
20. How important is it to be doing as well or better financially compared to other people in the population?
21. Relationships

    This section looks at relationships
22. How important is it to be connected to and have relationships with other people including family, friends, colleagues and romantic partners?
23. How important is it to have good family relationships?
24. How important is it to have good friendships?
25. How important is it to have good romantic relationships?
26. How important is it to have good relationships with colleagues at work?
27. How important is it to be able to communicate with government organisations, businesses or professionals?
28. How important is it to avoid social isolation? Social isolation is a lack of connections with or being cut off from other people. It can lead to loneliness.
29. Community Life

    This section looks at being involved with the local community
30. How important is it to be involved with the local community?
31. How important is it to be able to take part in community leisure activities such as sports clubs or a local choir?
32. How important is it for adults to support those around them such as by volunteering or doing charity work? Being involved with a community because it’s a good thing to do.
33. How important is it to be able to be involved in a religious or spiritual community if a person wishes?
34. How important is it to have a social identity? This means a feeling of belonging to a particular group.
35. Lifestyle

    The last section looks at lifestyle.
36. How important is it to have good personal maintenance? This is a person being able to look after themselves in a way that maintains their health and ability to live independently. It includes areas like personal hygiene.
37. How important is being able to make positive lifestyle choices such as being able to exercise?
38. How important is it to be able to avoid risky behaviours which can impact health and well-being in the short and long term? This includes smoking and drug abuse.
39. What else is important?

    You have worked through all of our suggestions for the social aspects of life that are needed to help adults who had cancer as a child lead a good life. 

    We are now interested in whether you have any other ideas of what these aspects might be. Is there anything we have missed?

    **In the box below, please add your comments about any other areas you think are important
    Free text box**

Thank you for completing the questionnaire

We will be in touch soon with the next round

END

**ROUND 2 QUESTIONNAIRE**

WELCOME

Thank you very much for your responses in the first survey.

It was clear that many people spent a lot of time on their answers and we are very grateful for this.

We appreciate that the first survey felt quite long to some of you- this survey is much shorter. 

This survey will summarise what everybody thought in the first survey and, after seeing this, ask if you want to change your answers in some areas.

RESULTS FROM ROUND 1

The first survey found that, on average, participants agreed 22 out of 34 categories relating to the social health of childhood cancer survivors are **important or very important**. This means we would include them in any future definitions of social health and use them to guide further research areas.

Because these categories have now been identified as important, you will not be asked anymore questions about them in this round. 

The categories are:

*Education*

- Receiving an education
- Completing school up to age 18
- Completing vocational training
- Having good school attendance
- Having enjoyed school
- Having participated in extra-curricular activities

*Independence and Autonomy*

- Being able to live independently
- Having autonomy
- Being able to undertake big responsibilities e.g. raising children

*Work and Finances*

- Having financial stability
- Having a job
- Having a well-paid job
- Having job satisfaction
- Having a job that builds skills

*Relationships*

- Having relationships with other people
- Having good family relationships
- Having good friendships
- Having good work relationships
- Being able to avoid social isolation

*Lifestyle*

- Being able to undertake personal maintenance
- Being able to make positive lifestyle choices
- Being able to avoid risky health behaviours

If you have any comments about these results, please write them in the free text box below

*Free text box*

RESULTS FROM ROUND 1 CONTINUED

The results also showed that, in general, people felt the important categories of social health should be the same for **adults who had cancer as a child**and the**general public**. 

Most people felt that what is different for childhood cancer survivors is the challenges they might face within each category and/or what success looks like to them.

This finding was noted in both the scores people gave each category and in the comments that they made throughout the survey.

Thank you to everyone for answering each question for childhood cancer survivors and the general public as it allowed us to demonstrate this.

If you have any comments about these results, please write them in the free text box below

*Free text box*

RESULTS FROM ROUND 1 AND ROUND 2 GUIDANCE

Finally, there were 12 areas of social health where there was more disagreement about whether these were **important** **or unimportant.**

As a result, you are now going to be asked about these areas again to see if we can come to a closer agreement about whether these categories are **important** **or unimportant**.

For the next questions:

1.  Please look at your previous answer (shown at the start of each question in this survey) and see how it compares to the average answer from the whole group of participants.

2.  Next, please read the comments that were based on everyone's answers in the last round. These are in no particular order with all comments mixed together.

3.  Finally, please rate the area again. You can change your rating or keep it the same. Please add any reasons for why you have changed your answer or kept it the same. This will help us to understand your answers and prepare for the next round of questions.

The rating scale is the same as the last round: **1** is **NOT at all important** and **7** is **VERY important**.

The areas come up in a different order to the last round.

ROUND 2 QUESTIONS

For the following twelve questions, after the round 1 comments, there is an individual 7-point Likert scale with a free-text box under each when viewed on the survey host website: [www.Onlinesurveys.ac.uk](http://www.Onlinesurveys.ac.uk).

See Appendix 2 for example as seen on Online Surveys.

1. **How important is it to be a homeowner? (R)**Participant is shown individualised Likert scale displaying the median score of all participants and their own score from the previous round.
    **In round 1, people commented that:**

- Being a homeowner is a personal choice that is an important life goal for some, but not others
- Renting and home ownership each have their own advantages and disadvantages, but both provide independence
- Being a homeowner can provide a sense of accomplishment which can be heightened if you have overcome challenges such as childhood cancer
- Some people see home ownership as a financial investment which provides more financial security in the long term

**Please re-rate your answer and add any reasons for why you have changed your rating or kept it the same.**7-point Likert scale and free text box

1. **How important is it to be doing as well or better financially compared to other people in the population?** **(S)**
   Participant is shown individualised Likert scale displaying the median score of all participants and their own score from the previous round.

   **In round 1, people commented that:**

- Looking at your own life and matching up your wants and needs to your finances is more important than comparing those to other people
- It is better to make sure everyone has what they need
- Health, happiness and life satisfaction not related to money is more important
- For some people, doing better financially than others might be seen positively as a marker of their abilities or as an achievement
- Following childhood cancer, doing as well or better financially than others might seem more important as it could be an indicator that cancer hasn’t held them back

**Please re-rate your answer and add any reasons for why you have changed your rating or kept it the same.**7-point Likert scale and free text box

1. **How important is it to be able to save money for luxuries?**This might include meals out, holidays abroad or a better car than necessary **(M)**

Participant is shown individualised Likert scale displaying the median score of all participants and their own score from the previous round.

**In round 1, people commented that:**   

- Everyone should have the knowledge, skills and ability to save money, but is up to each individual how they spend that money. It doesn’t have to be on a perceived ‘luxury’
- Time with family and friends, in any context, is more important than spending money on physical luxuries such as a car
- Following childhood cancer, luxuries such as holidays can give people a sense of making up for lost time and make them feel more ‘normal’ again
- Survivors may be more aware of life being short and the risk of long term effects from their cancer or treatment, making them keener to spend money on luxuries that help them enjoy life
- Saving for luxuries can be more difficult to achieve for childhood cancer survivors because they may be more likely to experience financial instability due to the impact of cancer across multiple areas of life
- Saving for luxuries may become more important to people as they get older

**Please re-rate your answer and add any reasons for why you have changed your rating or kept it the same.**7-point Likert scale and free text box

1. **How important is it to be involved in the local community? (AA)**

Participant is shown individualised Likert scale displaying the median score of all participants and their own score from the previous round.  **In round 1, people commented that:**       

- Being involved with the local community is important to some people but not others. How important this is depends on how well someone’s personality and interests match the needs of the local community
- Being involved in the local community can help people to connect with others and build friendships and support networks which provide a sense of belonging
- Community involvement can have wider, positive impacts e.g. it might make the community safer, it might lead to better facilities for everyone etc
- To be fully involved members of a community, survivors of childhood cancer might need extra support because of physical or cognitive problems
- For some childhood cancer survivors, particularly those with fewer personal relationships, not being involved in the local community may risk social isolation

**Please re-rate your answer and add any reasons for why you have changed your rating or kept it the same.**7-point Likert scale and free text box

1. **How important is it to be able to take part in community leisure activities such as sports clubs or a local choir? (BB)**Participant is shown individualised Likert scale displaying the median score of all participants and their own score from the previous round.  **In round 1, people commented that:**

- Being involved in community activities is important to some people but not others. How important being involved is depends on how well someone’s personality and interests match the activities available
- Being involved in community activities makes you more aware of others in your community
- Being involved in community activities benefits communities as a whole
- Childhood cancer survivors may find it more difficult to access activities, but should be supported to become involved with any activities they are interested in

**Please re-rate your answer and add any reasons for why you have changed your rating or kept it the same.**7-point Likert scale and free text box

1. **How important is it for adults to support those around them such as by volunteering or doing charity work?** **(CC)**
   Participant is shown individualised Likert scale displaying the median score of all participants and their own score from the previous round.

   **In round 1, people commented that:**

- Supporting others through voluntary work can help prepare survivors for other kinds of work
- Supporting others encourages socialising and prevents isolation
- It is important for childhood cancer survivors to feel like they are ‘giving back’, which may include to the charities that supported them as a patient
- Charities and the people they help benefit from the personal knowledge and experiences of childhood cancer survivors
- It might be important to support other people who have been in similar circumstances e.g. helping current patients with their cancer journey
- Supporting others can be time consuming

**Please re-rate your answer and add any reasons for why you have changed your rating or kept it the same.**7-point Likert scale and free text box

1. **How important is it to be able to be involved in a religious or spiritual community if a person wishes? (DD)**

Participant is shown individualised Likert scale displaying the median score of all participants and their own score from the previous round.

**In round 1, people commented that:**     

- Religious/ spiritual communities can help individuals to form a support network and provide sources of interaction with others
- Having a religious/ spiritual aspect to your life can offer hope, peace and emotional well-being during difficult times, including coping with long term effects of cancer
- Religious/ spiritual communities can be a source of guidance
- A cancer diagnosis as a young person might make you consider religion and spirituality more

**Please re-rate your answer and add any reasons for why you have changed your rating or kept it the same.**7-point Likert scale and free text box

1. **How important is it to have a social identity?**This means a feeling of belonging to a particular group **(EE)**

Participant is shown individualised Likert scale displaying the median score of all participants and their own score from the previous round.

**In round 1, people commented that:**       

- Cancer can make you feel you are defined by your cancer and that your identity has been taken away. Having a social identity can help you to feel part of something again and be known for the reasons you want rather than cancer
- Cancer might make you better at mixing with lots of groups of people because of periods away from your peers during treatment. Because of this you might not feel the need to belong to a single group with a specific social identity
- Having a social identity prevents isolation
- A social identity could be damaging if you only identify with a small community that alters your sense of self

**Please re-rate your answer and add any reasons for why you have changed your rating or kept it the same.**7-point Likert scale and free text box

1. **How important is being able to complete higher education e.g. getting a degree at university or similar? (D)**

Participant is shown individualised Likert scale displaying the median score of all participants and their own score from the previous round. See example in Appendix 1.

**In round 1, people commented that:**  

- Having options and being able to choose what you want to do after finishing school is very important. This includes university and apprenticeships
- Whilst university is crucial for people who want a career that requires a degree, many careers don’t require this
- An apprenticeship or working your way up in a job can lead to the same career and life success as a university degree
- University is about more than just education. It can help to build life skills, social skills and friendships etc.
- There may be increased barriers to completing higher education for those who had cancer as a child because of missed educational time in earlier years and/or the ongoing impacts of cancer
- University can be very expensive, leaving people with a lot of debt

**Please re-rate your answer and add any reasons for why you have changed your rating or kept it the same.**7-point Likert scale and free text box

1. **How important is it to achieve or have achieved good exam grades?** **(F)**

Participant is shown individualised Likert scale displaying the median score of all participants and their own score from the previous round.  **In round 1, people commented that:**     

- Good exam grades are not always necessary, people can be successful without them
- Each person should be supported to maximise their own potential which might not include good exam grades- working hard and doing your best can be more important than an exam grade itself
- The importance of exam grades is dependent on what each person wants to do following school e.g. they might be very important for someone wanting to go to university or other future opportunities
- For some people, passing an exam to allow them to get to their next goal is more important than how well that grade is achieved
- Following childhood cancer, getting back into school and sitting exams is a significant achievement in and of itself which should be recognised as much as the grade
- Some childhood cancer survivors may have cognitive problems after treatment making grades less important and other skills that develop long-term independence much more important
- Good exam grades can provide a concrete sense of achievement
- Too much emphasis on exam grades can be damaging to young people

**Please re-rate your answer and add any reasons for why you have changed your rating or kept it the same.**7-point Likert scale and free text box

1. **How important is it to have good romantic relationships? (W)**

Participant is shown individualised Likert scale displaying the median score of all participants and their own score from the previous round.
 **In round 1, people commented that:**

- Finding someone to connect with romantically is essential for some people, but not every person wants or needs a romantic relationship. Other kinds of relationship that provide support are more important for some people
- If romantic relationships are important to a person, they can enhance health and wellbeing
- The physical and psychological consequences of cancer can affect the ability to have romantic relationships. This can be very distressing if romantic relationships are important to that person

**Please re-rate your answer and add any reasons for why you have changed your rating or kept it the same.**7-point Likert scale and free text box

1. **How important is it to be able to communicate with government organisations, businesses or professionals? (Y)**

Participant is shown individualised Likert scale displaying the median score of all participants and their own score from the previous round.

**In round 1, people commented that:**

- Being able to communicate effectively with formal organisations or have support to do this is a necessary part of adult life. It allows people to access the right services, support and/ or benefits that they need.
- Childhood cancer survivors are more likely to need extra support from formal organisations. It is therefore important that they have the ability and confidence to communicate with these organisations, yet may face difficulties with this e.g. because of cognitive, hearing or mobility problems. Consequently, it is essential to support survivors with their formal communication

**Please re-rate your answer and add any reasons for why you have changed your rating or kept it the same.**7-point Likert scale and free text box

WHAT ELSE IS IMPORTANT?

At the end of the first survey everyone was asked if they could think of any other aspects of social health which they thought hadn't been covered. 

Below are 5 final questions covering topics suggested by yourselves as participants.

Please rate these in the same way as the other questions. 

For the following five questions, there is an individual 7-point Likert scale for adults who had cancer as a child with a free-text box under each when viewed on the survey host website: [www.Onlinesurveys.ac.uk](http://www.Onlinesurveys.ac.uk)

1. **How important is it to be able to have sexual relationships?**This was suggested separately to romantic relationships. **(FF)**
2. **How important is it to be able to access peer support?**This might include support groups or charities providing activities designed for those who experienced childhood cancer **(GG)**
3. **How important is it to be able to drive?**  **(HH)**
4. **How important is it to be able to access and utilise public transport?**This might include buses, trains and trams **(II)**
5. **How important is it to be able to express yourself creatively?**This might include through music, art, cooking or writing etc**. (JJ)**

THANK YOU

You have now completed the second round of the study.

Thank you very much for taking part.

We will be in touch soon with information on the next round.

**Round 3 Questionnaire**

WELCOME

Thank you very much for your responses in the second survey.

It was really helpful to see so many comments about why everyone did or didn't make changes to their original scores.  

We now have a list of different areas of social health grouped according to how important people found them.  

In this final round, we will:

- Ask you to look at three remaining areas of social health where there was disagreement about how important they are.
- Tell you what areas the group thought were most important and, from these, ask you to pick the three you think are most important.

ROUND 3 GUIDANCE

In round two, there were three areas of social health, that were asked about for the first time, where there was disagreement about how important they are. 

As a result, you are now going to be asked about these areas again to see if we can come to a closer agreement about whether these categories are **important** **or unimportant**. They will be presented in the same way as the second round.

For the next questions:

- Please look at your previous answer (shown at the start of each question in this survey) and see how it compares to the average answer from the whole group of participants.
- Next, please read the comments that were based on everyone's answers in the last round. These are in no particular order with all comments mixed together.
- Finally, please rate the area again. You can change your rating or keep it the same. Please add any reasons for why you have changed your answer or kept it the same. 

  The rating scale is the same as the other rounds: **1** is **NOT at all important** and **7** is **VERY important**.

ROUND 3 QUESTIONS

For the following three questions, after the round 2 comments, there is an individual 7-point Likert scale with a free-text box under each when viewed one the survey host website: [www.Onlinesurveys.ac.uk](http://www.Onlinesurveys.ac.uk).

See Appendix 2 for example as seen on Online Surveys.

1. **How important is it to be able to have sexual relationships? (FF)**Participant is shown individualised Likert scale displaying the median score of all participants and their own score from the previous round.  **In round 2, people commented that:**

- Sexual relationships are an important part of healthy relationships for many people but not everyone. The most important aspect is being able to have sexual relationships if a person wants them
- Some people may struggle with aspects of sexual relationships because of physical late effects and/ or sexual confidence following cancer treatment
- Sexual relationships can help people feel normal again following cancer treatment
- Some people felt that, in a romantic relationship, emotional intimacy was more important than physical intimacy

**Please re-rate your answer and add any reasons for why you have changed your rating or kept it the same.**7-point Likert scale and free text box

1. **How important is it to be able to drive? (HH)**Participant is shown individualised Likert scale displaying the median score of all participants and their own score from the previous round.
    **In round 2, people commented that:**

- Driving promotes independence but for many people, the same can be achieved using public transport. It is the option and ability to learn to drive if someone wishes which is important
- If you have mobility issues e.g. following cancer treatment, that make public transport more difficult, being able to drive might be more important in order to achieve independence
- Driving is less important if you live in a big city with good public transport and more important for people living in rural areas without this service

**Please re-rate your answer and add any reasons for why you have changed your rating or kept it the same.**7-point Likert scale and free text box

1. **How important is it to be able to express yourself creatively? This might include through music, art, cooking or writing etc. (JJ)**Participant is shown individualised Likert scale displaying the median score of all participants and their own score from the previous round.  **In round 2, people commented that:**

- Creative expression provides an opportunity for escapism which is important for good health and well being
- Depending on the nature of the expression, it might provide opportunities to socialise or even career opportunities
- Not all people want or need a creative outlet but for those that do, being able to engage in something that makes them happy is very important, particularly following cancer treatment
- Any outlet could be beneficial, be that creative or practical

**Please re-rate your answer and add any reasons for why you have changed your rating or kept it the same.**7-point Likert scale and free text box

RESULTS REVIEW

From the first two rounds of the study we have generated a list of main categories and sub-categories of social health which have been grouped according to how importantly they were rated.

**Main categories**

**The most important main categories are:**

Education

Independence and autonomy

Work and Finances

Relationships

Lifestyle

Community life was found to be less important overall.

**Sub categories**

**Most important:**

*Education*

- Completing school up to age 18
- Completing vocational training
- Having good school attendance
- Having enjoyed school
- Having participated in extra-curricular activities

*Independence and autonomy*

- Being able to live independently
- Having autonomy
- Being able to undertake big responsibilities e.g. raising children
- Being able to use public transport

*Work and finances*

- Having financial stability
- Having a job (paid or unpaid)
- Having a well-paid job
- Having job satisfaction
- Having a job that builds skills

*Community Life*

Being able to access peer support e.g. through a support group

*Relationships*

Having good family relationships

Having good friendships

Having good work relationships

Being able to avoid social isolation

*Lifestyle*

- Being able to undertake personal maintenance
- Being able to make positive lifestyle choices
- Being able to avoid risky health behaviours **​**

**Quite important:**

*Education*

- Completing higher education
- Good exam grades

*Work and finances*

Saving money for luxuries 

*Relationships*

Good romantic relationships

Being able to communicate with formal organisations

*Community life*

Being able to take part in community activities

Being part of religious or spiritual community

Having a social identity 

**Neither important nor unimportant:**

*Work and finances*

Being a homeowner

*Community Life*

Supporting others e.g. through charity work 

**Not important:**

*Work and Finances*

Doing better financially than other people

Do you agree with these results? If you have any comments, please write them in the box below.

Free text box

WHAT IS MOST IMPORTANT?

Finally, we would like to know what you think is most important from the group of sub categories that were rated as most important during the survey.

Please look carefully through the list below and then select the **three** areas that you think are most important. The list has been randomised.

Tick box displayed next to each category. Three can be selected.

Being able to avoid social isolation

Being able to undertake big responsibilities

Having a job that builds skills

Having good school attendance

Having good work relationships

Having enjoyed school

Having good family relationships

Having a well-paid job

Having financial stability

Being able to undertake personal maintenance

Being able to avoid risky health behaviours

Having been able to participate in extra-curricular activities

Completing school up to age 18

Having good friendships

Having job satisfaction

Being able to complete vocational training

Being able to live independently

Having a job (paid or unpaid)

Being able to use public transport

Having autonomy

Being able to make positive lifestyle choices

Being able to access peer support e.g. through a support group

THANKYOU

You have now completed the final round of the study.

Thank you **very** much for taking part. We really value your insight and contribution.

We will update you with a final report of the results and how we are planning to use them.

If you would like to ask any questions in the mean-time, please email us at ysrccyp@leeds.ac.uk

**APPENDIX 1**

**Example of 1^st^ round question displaying 7-point Likert scale.**

**
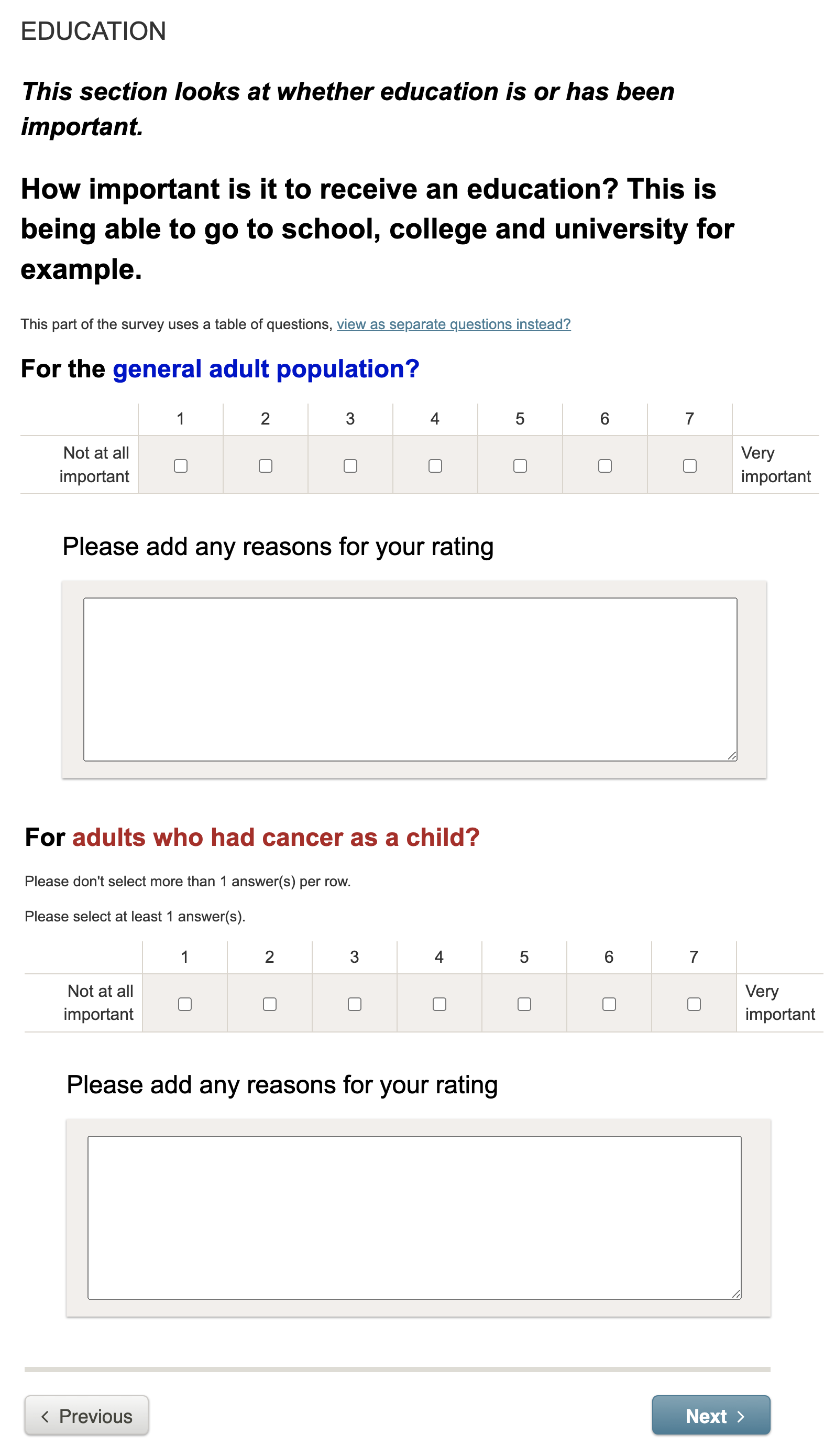
**

**APPENDIX 2**

**Example of 2^nd^ and 3^rd^ round question displaying personalised Likert scale**

**
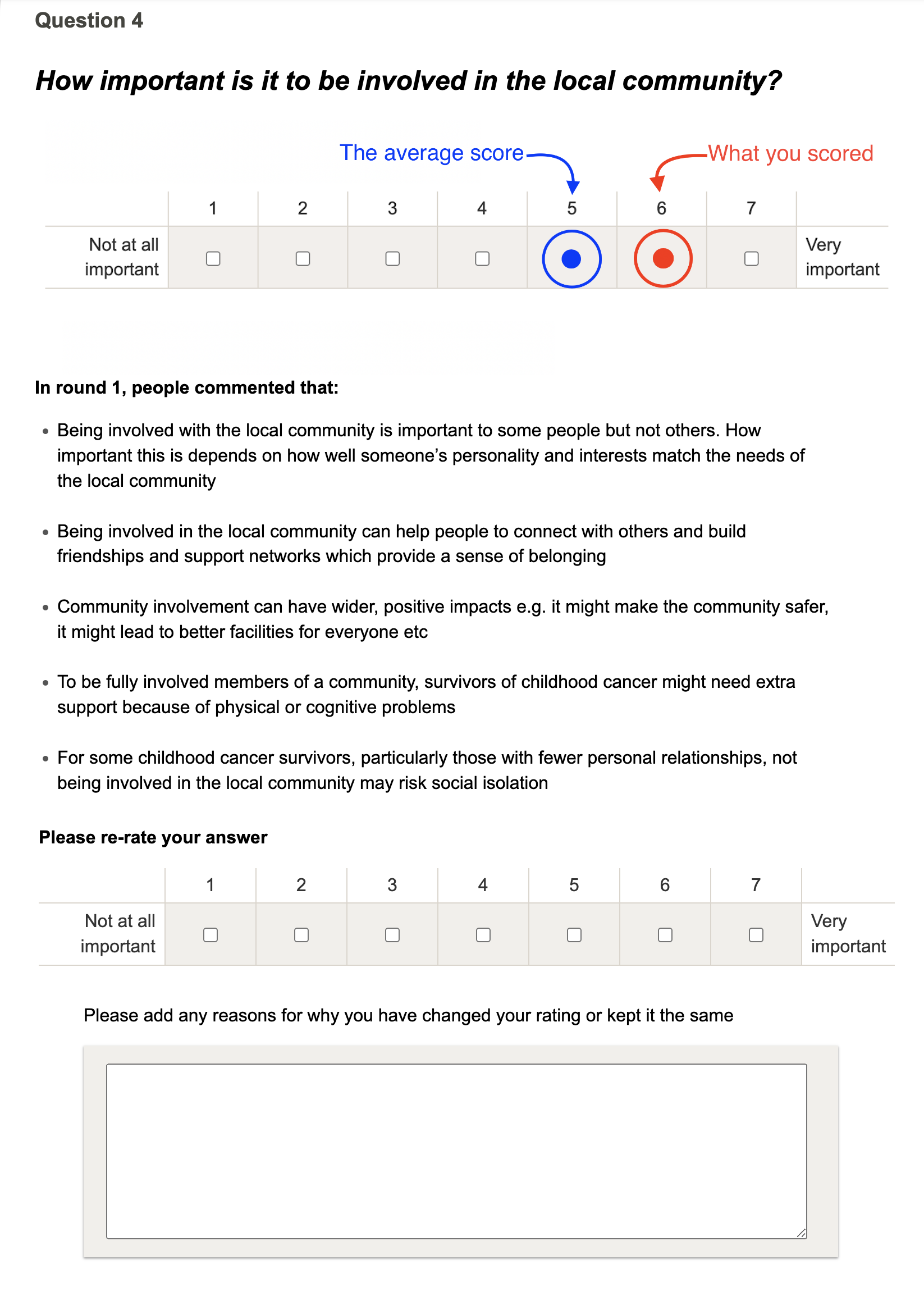
**
