# Supplementary material for: Identifying social outcomes of importance for childhood cancer survivors: an e-Delphi study
Source: J Patient Rep Outcomes. 2024 Feb 5;8:14. doi: 10.1186/s41687-023-00676-7 (PMC10844160; doi:10.1186/s41687-023-00676-7)
Supplement: Supplementary file 2 — Additional file 2: Qualitative results overview. [file 41687_2023_676_MOESM2_ESM.docx]

# Identifying Social Outcomes of Importance for Childhood Cancer Survivors: An e-Delphi Study. Supplementary File 2.

**QUALITATIVE RESULTS OVERVIEW**

Themes from free-text comments.

1. **Social outcomes for the general public and childhood cancer survivors ought to be the same.**
   - A few areas were suggested as potentially being more important to survivors:
     - Being able to communicate with government organisations, businesses or professionals. Survivors are more likely to need support from these organisations e.g. disability benefits.
     - Being a homeowner or financially successful might provide a greater sense of accomplishment as they can feel like a marker that cancer hasn’t held a person back.
     - Being involved in the local community might reduce the risk of social isolation, particular for survivors with fewer personal relationships due to the effects of disease and/or treatment.
     - Being able to drive might be more important if a survivor has a physical impairment following treatment that makes public transport difficult.
     - Being able to make positive lifestyle choices given the long-term physical health implications of disease and treatment.
2. **A successful social outcome is unique to each individual and might be altered following disease or treatment.**

Examples:

| Education | - Following childhood cancer, getting back into school and sitting exams is a significant achievement in and of itself which should be recognised as much as the grade. - Some childhood cancer survivors may have cognitive problems after treatment making grades less important and other skills that develop long-term independence much more important. - Time frames and settings may need adjustment to ensure survivors can reach their potential. |
| --- | --- |
| Independence and Autonomy | - A successful outcome for an individual with significant long-term impacts may not be complete independence but instead focuses on realistic elements of independence. |
| Work and Finances | - Time frames for anticipated work/ financial goals may need adjustment. - Following childhood cancer, luxuries such as holidays can give people a sense of making up for lost time and make them feel more ‘normal’ again. - A survivor might have additional needs causing a financial burden not experienced by the general population. These may not be fully covered by grants, disability allowances etc. |
| Relationships | - Family relationships may become closer during and following treatment due to the lived experience of childhood cancer as a family. - Breaks in social integration during treatment can push survivors into being more self-reliant and less interested in a high volume of friendships/ other relationships. - There may be a greater need for separate groups of friends including ‘cancer friends’ who understand what an individual has been through and ‘other friends’ who provide space away from the cancer experience. |
| Community Life | - Cancer might make a person better at mixing with lots of groups of people because of periods away from peers during treatment. Because of this they might not feel the need to belong to a single group with a specific social identity. - Charity work/ volunteering, particularly with relevant charities may become more important if survivors experience a sense of wanting to ‘give back’. |
| Lifestyle | - Survivors may feel a great desire to look after their body, make positive lifestyle choices and avoid risky health behaviours. This can stem from knowledge and ongoing education about long term impacts and from a desire to retain control over their body. - Survivors may want to engage in more risk-taking behaviours e.g. extreme sports because they feel invincible following cancer. |

1. **Childhood cancer survivors being able to make their own choices about their life is important. Cancer should not take away options or opportunities.**

Examples:

| Education | - Ensuring that childhood cancer survivors have the option to go on to further/ higher education if this is something they want. - Providing opportunities for involvement in extracurricular activities if wished is important to allow building of other skills and social reintegration. |
| --- | --- |
| Independence and Autonomy | - Not everyone wants to live independently but if they do, this should be supported where possible. - Some people prefer to have others take over/ support medical decisions rather than acting alone. - Big responsibilities such as raising a child should be available to all if they want it but individuals should also be respected for having their own goals. Wanting a particular responsibility should not be assumed. |
| Work and Finances | - Having employment options and being supported to achieve career aspirations. - Being able to have a job that builds skills provides greater choices across adult life, further than work alone. - Having choices about finances and being able to act on them helps foster feelings of accomplishment and fulfilment e.g. being able to save money for luxuries or a house. |
| Relationships | - Not all people want or need romantic/ intimate relationships. Other kinds of supportive relationship are more important to some people. It is critical that cancer survivors can have these relationships if they wish. |
| Community Life | - Being involved with the local community is important to some people but not others, as for the general public. How important this is depends on how well someone’s personality and interests match the needs of the local community. - Having a religious/ spiritual aspect to life can offer hope, peace and emotional well-being during difficult times, including coping with long term effects of cancer. Cancer survivors should have the choice to access a religious community if they wish. |
| Lifestyle | - Having knowledge about the long-term risks associated with childhood cancer and its treatment is key to ensuring survivors can make informed lifestyle decisions. |

1. **There are barriers to social success faced by childhood cancer survivors**Examples:

| Education | - Missed educational time due to treatment or late effects. - Cognitive and physical impacts of disease and/or treatment. These may impact mandatory education, higher/ further education and the ability to take part in extra-curricular activities. - When a family may have faced significant financial burden due to the cancer diagnosis, expenses for higher education and/or other activities can be too great. |
| --- | --- |
| Independence and Autonomy | - Cognitive and physical impacts may be barriers to independent living, driving and accessing public transport. - Cancer treatment may affect ability to take on big responsibilities such as having children. An awareness of potential risks and issues is very important. - Reliance on others during cancer treatment can persist and act as a barrier to full independence. |
| Work and Finances | - Physical and cognitive impacts may reduce access to work that could build skills. Lack of opportunity for fulfilling work creates a cycle of negative impacts on physical, mental and social wellbeing which further impact ability to find satisfying work. - Saving money for luxuries may be more difficult because of financial instability as a result of the physical/ cognitive impacts of disease/ treatment |
| Relationships | - Physical consequences of cancer may impact romantic and sexual relationships e.g. because of previous surgery or radiotherapy. - Psychological consequences such as confidence issues creating barriers to friendships, romantic and sexual relationships. - Lack of understanding from peers and time out of school can be barriers to friendships during education. - Cognitive, visual, hearing and mobility problems could all generate barriers to effective formal communication e.g. in the work place or with government organisations or professionals in other roles. |
| Community Life | - Physical and cognitive problems might affect ability to access and or fully take part in some activities without extra support. - Impacts on confidence may prevent a survivor taking part in activities they are interested in. - Cancer can make people feel solely defined by their disease. It can be difficult to find their own social identity again. |
| Lifestyle | - Physical impacts e.g. limb surgery can make it harder to exercise to maintain health. - Cognitive impacts can make it harder to understand health risks of treatment and make positive lifestyle choices. |
